# Exhaled Aerosols in SARS-CoV-2 Polymerase Chain Reaction-Positive Children and Age-Matched-Negative Controls

**DOI:** 10.3389/fped.2022.941785

**Published:** 2022-07-18

**Authors:** Desiree Gutmann, Helena Donath, Laura Herrlich, Timon Lehmkühler, Anton Landeis, Emily R. Ume, Martin Hutter, Ann-Kathrin Goßmann, Frederik Weis, Maximilian Weiß, Holger F. Rabenau, Stefan Zielen

**Affiliations:** ^1^Division of Allergology, Pulmonology and Cystic Fibrosis, Department for Children and Adolescents, University Hospital Frankfurt, Goethe University, Frankfurt, Germany; ^2^Palas GmbH, Partikel- und Lasermesstechnik, Karlsruhe, Germany; ^3^Institute for Medical Virology, University Hospital Frankfurt, Goethe University, Frankfurt, Germany

**Keywords:** exhaled aerosol, COVID-19 in children, COVID-19, aerosols, acute respiratory tract infection

## Abstract

**Background:**

Children and adolescents seem to be less affected by the severe acute respiratory syndrome coronavirus 2 (SARS-CoV-2) disease in terms of severity, especially until the increasing spread of the omicron variant in December 2021. Anatomical structures and lower number of exhaled aerosols may in part explain this phenomenon. In a cohort of healthy and SARS-CoV-2 infected children, we compared exhaled particle counts to gain further insights about the spreading of SARS-CoV-2.

**Materials and Methods:**

In this single-center prospective observational trial, a total of 162 children and adolescents (age 6–17 years), of whom 39 were polymerase chain reaction (PCR)-positive for SARS-CoV-2 and 123 PCR-negative, were included. The 39 PCR-positive children were compared to 39 PCR-negative age-matched controls. The data of all PCR-negative children were analyzed to determine baseline exhaled particle counts in children. In addition, medical and clinical history was obtained and spirometry was measured.

**Results:**

Baseline exhaled particle counts were low in healthy children. Exhaled particle counts were significantly increased in SARS-CoV-2 PCR-positive children (median 355.0/L; range 81–6955/L), compared to age-matched -negative children (median 157.0/L; range 1–533/L; *p* < 0.001).

**Conclusion:**

SARS-CoV-2 PCR-positive children exhaled significantly higher levels of aerosols than healthy children. Overall children had low levels of exhaled particle counts, possibly indicating that children are not the major driver of the SARS-CoV-2 pandemic.

**Trial Registration:**

[ClinicalTrials.gov], Identifier [NCT04739020].

## Introduction

The severe acute respiratory syndrome coronavirus 2 (SARS-CoV-2) and the associated pulmonary disease [coronavirus disease–2019 (COVID-19)] have caused millions of deaths worldwide (1). Since the virus’ first description in December 2019, the pandemic has driven countries all around the globe to install various non-pharmaceutical interventions (NPIs) to mitigate the transmission of the virus. Prior to the global spread of the omicron variant, children and adolescents seemed to be less affected by the disease in terms of both, case numbers and severity (2, 3). However, the NPIs often included closing of schools, kindergartens and other educational and recreational groups for children and adolescents, which had a great impact on children’s mental health and development (4).

In line with other viral illnesses, the symptomatology of a SARS-CoV-2 infection differs between children and adults. The most common presentation of COVID-19 in adults include early anosmia, followed by fever, dry cough and dyspnea (5–8). However, especially in older patients, it can cause severe pneumonia, which can lead to acute respiratory failure and fulminant sepsis with thromboembolic complications (5, 6). Children were more often found to be asymptomatic or mildly affected (9, 10). If symptoms were present, usually, headache, fatigue, fever, cough and sore throat were predominant, while hypoxemia and dyspnea remained rare (9–11). In general, illness duration was reported as shorter in children than in adults (10).

SARS-CoV-2 is spread through three main routes: (1) droplets, (2) aerosols, and (3) fomites (12–14). However, as per current research, the latter plays a lesser role in the spread of the virus (15–17). In contrast, aerosols seem to be a primary transmission route, as so called “super spreader” events describe clusters of cases especially in close proximity, but not necessarily with direct contact to an infected individual (16, 18–21). In the past, aerosols have been found to be important transmission routes for other bacteria and viruses; e.g., mycobacterium tuberculosis, influenza viruses and respiratory syncytial viruses (RSV) (22–25). In general, the term “aerosol” describes a suspension of particles with a gas (12, 26). According to the world health organization (WHO) definition, aerosols are suspensions containing particles <5 μm, while droplets contain particles >5 μm (27). However, bigger “droplet nuclei” can also evaporate into multiple smaller particles (27, 28).

In the mucosa of the upper respiratory tract, shear stress and airflow lead to surface instability, which breaks the fluid lining into small droplets and generates aerosols (29). In the lower respiratory tract, the surface instability stems from closing and reopening of collapsed terminal airways during tidal breathing, which also leads to the formation of small particles, exhaled as aerosols (30). Every human exhales a baseline amount of aerosols during tidal breathing, which is increased during speech, laughter or singing (31, 32). Certain infections might also increase the exhaled particle count, as some studies could show an increase in exhaled particles with SARS-CoV-2 infection in primates and individual humans (33, 34). When comparing exhaled particles between adults and adolescents, similar ranges for speaking were observed, but adolescents showed lower values when singing (35). Generally, it appears that exhaled particle counts increase with age (34), which could indicate that children pose a lower risk in viral transmission *via* aerosols (17). This might be due to the immature airway structure of children, which is composed of fewer alveoli and terminal bronchioles, as well as smaller breathing volumes and lower exhaled air speed (36, 37). Additionally, the cough push is less intensive in children compared to adults. However, the baseline exhaled particle count and changes secondary to respiratory tract infections are not well studied in children and adolescents.

As mentioned above, children and adolescents seem to be less affected by COVID-19, which appears to be due to (1) lower susceptibility to the virus and (2) a less severe disease course. The former might be explained with lower Angiotensin-Converting Enzyme 2 (ACE 2) expression in the mucosa, which leads to less entry-points for the virus (3, 38, 39). The latter is apparent through the high number of mild and asymptomatic cases, which often are only detected as incidental findings from screening tests (3, 40). This prospective study investigated the baseline level of exhaled particle count in healthy and SARS-CoV-2 infected children.

## Materials and Methods

### Study Design

In a prospective observational cohort study, exhaled aerosol concentration and particle size were measured in SARS-CoV-2 polymerase chain reaction (PCR)-positive children and age matched healthy controls in the rhine main area and the city of Frankfurt, from January 18th to December 31st, 2021. Eligible participants were children and adolescents (6–17 years) with a SARS-CoV-2 PCR test prior to aerosol measurement.

Before recruitment into the study, detailed verbal and written information was provided for all participants and their legal guardians. With all patients and caregivers, the aims and risks of the study were discussed in detail. Prior to the start of the measurements, written consent, and, if appropriate, assent was obtained from all participants and their legal guardians. The investigation was approved by the Ethics Committee of the Goethe University Frankfurt (number 20–1001) and was registered under the ClinicalTrials.gov (Identifier: NCT04739020). The study was supported by a grant of Palas (Karlsruhe, Germany).

### Participants

The participants were recruited in two ways: Firstly, children admitted to the Children’s Hospital of the Goethe-University Hospital, Frankfurt, Germany, with SARS-CoV-2 PCR test, as part of the routine admission screening. Secondly, in cooperation with local physicians, SARS-CoV-2 PCR-positive children in the rhine main and Frankfurt city area were contacted, and, if the caregivers agreed, aerosol measurements were conducted in their homes. All participants were tested for SARS-CoV-2 *via* PCR prior to aerosol measurement. To account for differences between pre- and post-pubertal participants, it is a common practice to analyze pediatric data before and after the age of 12 years. Therefore, a sub-analysis in the age groups 6–11 and 12–17 years was conducted. Subjects were excluded from the study, if unable to participate in aerosol measurement, or if they and/or their legal guardian could not understand the extend and consequences of the study.

### Study Procedures

#### Medical and Clinical History

A standardized questionnaire was used to record medical history, including preexisting medical conditions and medications. In addition, to obtain a clinical history, all participants together with their guardians were asked about acute symptoms of a viral illness in the context of SARS-CoV-2 infections. Those symptoms included fever, cough, shortness of breath, loss of taste or smell, sore throat, muscle pain, diarrhea and vomiting.

For every participant, height and weight were recorded and the body mass index (BMI), as well as the weight-for-height z-score were calculated. The z-score represents the number of standard deviations above or below a certain reference mean or median value. It is recommended by WHO to use z-scores to compare weight and height in smaller children (41).

#### Aerosol Measurement

In order to measure the exhaled aerosols, an aerosol spectrometer was used to count and size the exhaled particles (Resp-Aer-Meter, Palas GmbH, Karlsruhe, Germany). Particles in the size range of 0.15–5.0 μm with sizing resolution of 16 channels/decade were detected. To count the particles, an optical sensor creates a defined optical measurement volume using a polychromatic light source. The exhaled particles travel through this volume, generating a scattered light pulse. From the quantity and intensity of this light pulse, the particles’ size and quantity can be determined.

For each measurement, a sterile sampling kit was used. The participants wore a nose clip, while breathing into a mouth piece with normal tidal breathing. The mouthpiece was connected with a T-piece, to a HEPA filter, which prevented particles from the surrounding air to mix with the exhaled particles, and to a heated hose, which was connected to the measurement device (Supplementary Figure 1A and 1B). After about 1 min of tidal breathing, the remaining ambient aerosols were washed out from the hose and measuring device and the participants exhaled particle measurement was started. Each measurement lasted for about 1–1.5 min. Afterward, the results were displayed as a graphical curve and in numerical form (Supplementary Figure 2).

#### Spirometry

Using a hand-held device (Asthma Monitor^®^ AM; VIASYS Healthcare GmbH, Höchberg, Germany), spirometry measurements were conducted with every participant according to the American Thoracic Society (ATS) and the European Respiratory Society (ERS) recommendations (42). The following parameters were obtained: Peak Expiratory Flow (PEF) and Forced Expiratory Volume in the first second (FEV1). To account for the influence of age, weight, and sex on FEV1, the FEV1% pred (FEV1 percentage of the predicted value) was calculated.

### Outcomes

Primary aims of this investigation were to compare the exhaled particle counts in SARS-CoV-2 PCR-positive and age-matched-negative children and adolescents and to measure the baseline exhaled particle count in healthy children and adolescents. As secondary outcomes, change in exhaled particle concentration with age, sex, lung function, height, weight, z-score and BMI were assessed and the possible correlation between symptoms of viral illness and exhaled particle concentration measurements were examined.

### Statistical Analysis

For statistical analysis, GraphPad Prism 5.01 (GraphPad Software, Inc.) and R 4.0.4 were used. All values were presented as median and range for numeric data and as percentage for count data.

The Wilcoxon-Mann-Whitney *U*-Test was used to test for group differences in numeric data and chi-squared test was used for count data. A *p*-value of < 0.05 was considered statistically significant.

The sensitivity and specificity of exhaled particle count measurements was assessed using Receiver Operating Characteristic (ROC) analysis. Correlations between cycle threshold (Ct) values and exhaled particle count measurements were calculated *via* Spearman correlation.

## Results

### Patient Characteristics

In total, 162 children and adolescents were analyzed. Of those, 39 participants were tested positive for SARS-CoV-2 *via* PCR and 123 participants were tested negative. The 39 PCR SARS-CoV-2 positive children and adolescents were compared to age-matched SARS-CoV-2 PCR-negative controls. Patient characteristics are shown in Table 1. Overall, the characteristics of the SARS-CoV-2 PCR-positive and age-matched -negative children were not statistically different. Both groups had the same median age of 12 years (*p* = 0.412) and had the same slight male predomination (59.0 vs. 41.0%). The median BMI and z-score in the SARS-CoV-2 PCR-positive group were lower than in the SARS-CoV-2 negative group, with 17.2 vs. 19.8 kg/m^2^ (*p* = 0.051) and −0.2 vs. 1.0 (*p* = 0.035), respectively.

**TABLE 1:** Characteristics of polymerase chain reaction (PCR) severe acute respiratory syndrome coronavirus 2 (SARS)-CoV-2 PCR-positive children and -negative age-matched controls.

	SARS-CoV-2 PCR-positive (*n* = 39)	SARS-CoV-2 PCR-negative (*n* = 39)	Total (*n* = 78)	*p*-value
**Sex**				
Female	16 (41.0%)	16 (41.0%)	32 (41.0%)	1.000
Male	23 (59.0%)	23 (59.0%)	46 (59.0%)	
**BMI (kg/m^2^)**				
Median	17.2	19.8	18.6	0.051
Range	12.1–29.7	13.5–32.4	12.1–32.4	
**Z-Score**				
Median	−0.2	1.0	0.1	0.035
Range	−4.2–1.9	−3.0–2.4	−4.2–2.4	
**Age (years)**				
Median	12.0	12.0	12.0	1.000
Range	6.0–17.0	6.0–17.0	6.0–17.0	
**FEV1 (%pred.)**				
Median	80.5	85.0	83.2	0.066
Range	31.1–169.8	42.0–156.0	31.1–169.8	
**Ct value**				
Median	22	>40.0		
Range	17.7–31.5			
**Comorbidities**				
None	33 (84.6%)	27 (69.2%)	60 (76.9%)	0.178
Allergy	3 (7.7%)	1 (2.6%)	4 (5.1%)	0.615
Diabetes	0 (0%)	3 (7.7%)	3 (3.8%)	0.240
Respiratory disease	2 (5.1%)	7 (17.9%)	9 (11.5%)	0.154
Neurological disease	1 (2.6%)	1 (2.6%)	2 (2.6%)	1.000

*p-Values for differences in SARS-CoV-2 PCR-positive and -negative participants are derived from Wilcoxon-Mann-Whitney U-Test for numeric data and from chi-square test for count data.*

### Medical and Clinical History

Pre-existing medical conditions were present in 15.4% (6/39) of participants in the SARS-CoV-2 PCR-positive group and 30.8% (12/39) in the SARS-CoV-2 PCR-negative group (Table 1). The most common pre-existing medical conditions were asthma (2.6 vs. 15.4%), diabetes (0 vs. 7.7%) and allergies (7.7 vs. 2.6%).

Overall, in the SARS-CoV-2 PCR-positive group, 48.7% (19/39) had typical symptoms of a viral illness. The following symptoms were reported: cough in 35.9% (14/39), loss of taste/smell and sore throat both in 15.4% (6/39), shortness of breath in 7.7% (3/39), fever in 5.1% (2/39), and muscle pain in 2.6% (1/39). No participants in this group reported diarrhea or vomiting as acute symptoms. In the SARS-CoV-2 PCR-negative group, 10.3% (4/39) reported acute symptoms: 5.1% (2/39) reported each diarrhea, vomiting and sore throat, 2.6% (1/39) reported cough. No participants in this group reported fever, shortness of breath, muscle pain or loss of taste/smell as acute symptoms.

### Aerosol Measurements

#### Exhaled Particle Count

When looking at the exhaled particle counts of all children and adolescents (SARS-CoV-2 PCR-positive and -negative), the median was 211/L (0–6955/L).

**FIGURE 1:**
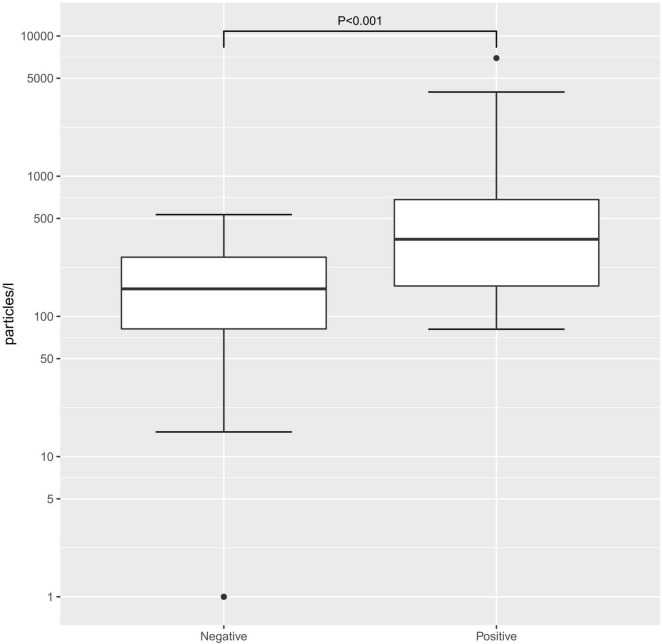
Exhaled particle counts in severe acute respiratory syndrome coronavirus 2 (SARS-CoV-2) polymerase chain reaction (PCR)-positive and -negative children. Exhaled particle counts in particles/L Exhaled particle counts in particles/L, displayed on a logarithmic scale.

When comparing the exhaled particle count in the SARS-CoV-2 PCR-positive and age-matched -negative groups, the median was significantly higher in SARS-CoV-2 PCR-positive children and adolescents (355/L [81–6955/L]) than in SARS-CoV-2 PCR-negative participants (151/L [1–533/L]; *p* < 0.001, Figure 1). When looking at the different age groups, in the group of children 6–11 years old, there was a significant difference between SARS-CoV-2 PCR-positive and -negative children, with a median exhaled particle count of 287/L (86–6955/L) and 145/L (0–533/L; *p* = 0.023), respectively. Furthermore, in the group of adolescents, 12–17 years old, a significant difference in exhaled particle counts could be found between the two groups (396.0/L [81–3982/L] vs. 171/L [0–502/L]; *p* = 0.004).

When separating the SARS-CoV-2 PCR-positive and -negative groups by age at a cut-off of age 12, within the two groups, there was no significant difference in exhaled particle counts. Within the PCR-positive groups, the median particle counts in children 6–11 years old and 12–17 years old were 287/L (86–6955/L) and 396/L (81–3982/L; *p* = 0.777), respectively. In the SARS-CoV-2 PCR-negative groups, the median particle counts in children 6–11 years old and 12–17 years old were 145/L (0–533/L) and 171/L (0–502/L; *p* = 0.955), respectively.

No significant difference of exhaled particle counts was found due to sex within the SARS-CoV-2 PCR-positive (*p* = 0.461) and -negative (*p* = 0.710) group. In addition, there was no correlation between exhaled particle counts and BMI (*p* = 0.370), z-score (*p* = 0.572), height (*p* = 0.189) or weight (*p* = 0.185).

**FIGURE 2:**
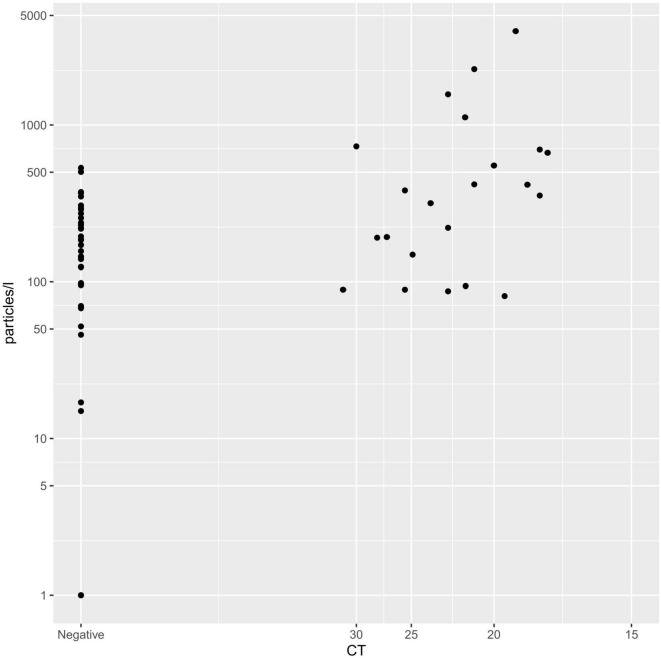
Spearman correlation of CT-value and exhaled particle count. Displaying the correlation between Ct-value (x-axis) and exhaled particles (y-axis).

When correlating the Ct value of each PCR test to the exhaled particle count measurements, there was a significant, negative correlation (Spearman correlation, r: −0.249; *p* < 0.001; Figure 2).

#### Receiver Operating Characteristic Analysis of Aerosol Concentration

**FIGURE 3:**
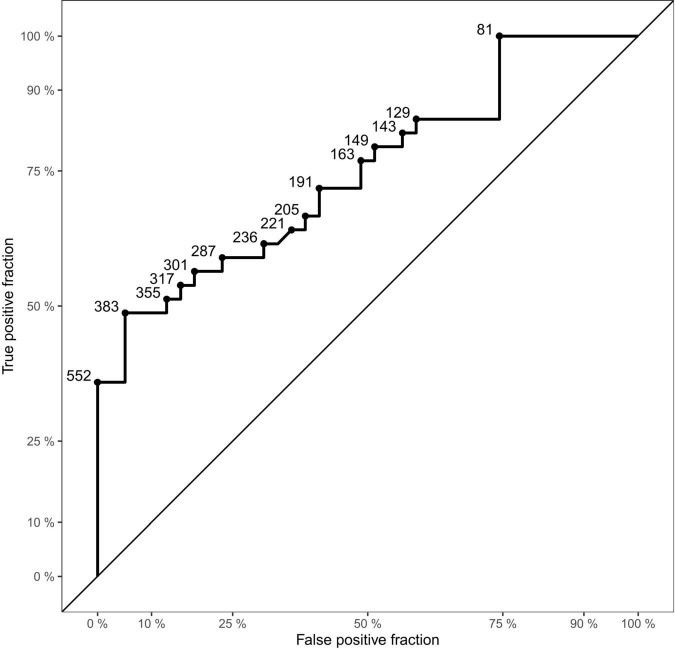
Receiver Operating Characteristic (ROC) curve of the dataset. Displaying sensitivity (true positive fraction) in the *y*-axis and 1-specificity (false positive fraction) on the *x*-axis. Points on the curve show examples of cut-off values (aerosol particles per liter).

A ROC analysis was conducted to determine the accuracy of the exhaled particle count measurements in detecting SARS-CoV-2 PCR -positive individuals (Figure 3). The analysis of all age-matched children showed an area under the curve (AUC) of 0.75. When separating the participants by age, the AUC was higher in adolescents, aged 12–17 years (AUC 0.76), compared to younger children, aged 6–11 years (AUC 0.73).

### Spirometry

In the spirometry measurements, there was no difference between the two groups. In the SARS-CoV-2 PCR-positive and -negative group, the median PEFs were 271 L/Min (106–505 L/Min) and 286 L/Min (123–505 L/Min; *p* = 0.961), respectively. The median FEV1s were 2.3 L/s (0.9–3.8 L/s) and 2.4 L/s (0.7–4.1 L/s; *p* = 0.270), respectively. In addition, the median FEV1% pred were 80.5% (31.1–169.8%) and 85.0% (42.0–156.0%; *p* = 0.066), respectively.

## Discussion

In general, transmission of bacteria and viruses *via* aerosols has been apparent for several years (24, 25). As demonstrated in various studies, aerosols are the primary transmission route of SARS-CoV-2 (16, 18–21). The virus was found to retain structural integrity for more than 12 h in aerosol suspensions (43). While children and adolescents have been reported to transmit the virus (2, 44, 45), they do not seem to be the main driver of viral spread, especially prior to the omicron wave of infections that started in December 2021; e.g., a transmission from adult to child has found to be more common than vice versa, in multiple settings (39, 46). Viral respiratory illnesses can spread rapidly from one child to another, for example, in a study from Chu et al. during RSV season, after the first case of RSV was found in a childcare group, within 1 week, the virus was transmitted to 50% of children who were in the same room (47). However, in the case of SARS-CoV-2, children seemed to be less contagious than adults (48).

One reason for decreased contagiousness is probably that children emit a lower number of exhaled particles. This might be due to anatomical differences in the immature airway structure of children, which consists of fewer alveoli and terminal bronchioles, which are thought to be the origin of aerosol production (36, 37). In line, the current study demonstrated that children and adolescents overall emitted only a small amount of exhaled particles when compared to adults (49). In addition, in all healthy controls (SARS-CoV-2 PCR-negative) a median exhaled particle count of 170.0/L (68.0–298.5/L) was measured. Edwards et al. found an increase in exhaled particles with age (34). In the present study, when the healthy controls were divided into age groups (cut-off 12 years), there was no difference between the two groups. The overall lower baseline exhaled particles might be contributing to the finding that children, sometimes even with higher viral loads than adults, seem to be less symptomatic and less contagious (3, 17, 40).

Another reason for possibly decreased contagiousness of children and adolescents might be decreased susceptibility to the virus and less severe symptoms (3, 48). As SARS-CoV-2 enters the human body through ACE 2 receptors, the lower expression of those receptors in children likely plays a role in the decreased susceptibility (3, 38, 39). In addition, an immunity against other seasonal coronavirus contributes to the multitude of asymptomatic cases (3, 40). Nevertheless, in coherence with our previous findings and the findings of Edwards et al., SARS-CoV-2 PCR-positive children and adolescents produced significantly more exhaled particles than SARS-CoV-2 PCR-negative controls (34, 49). In addition, we found a significant negative correlation of Ct value and exhaled particle count, showing higher exhaled particle counts in lower Ct values. While statistically significant, this increase of exhaled particle counts in SARS-CoV-2 PCR-positive children and adolescents was not as substantial, as seen in our previous work with adults (49). A possible explanation for this observation being lower rates of symptomatic participants, as only 48% of all SARS-CoV-2 positive children were symptomatic and those had only mild to moderate symptoms. However, the lower overall aerosol production in children and adolescents most likely contributes to this difference aswell.

Apart from the aforementioned correlation between exhaled particle counts and age, Edwards et al. also found the BMI to be a confounding factor (34). This correlation was not found in the present cohort, however, as BMI is not a very reliable measurement in children, height, weight and z-score were analyzed, but did also not show any relationship. Furthermore, there was no significant association with lung function.

The present findings suggest that measurements of exhaled particle counts do not have sufficient validity to be used as a testing tool in children (AUC 0.75). However, previous studies (35, 46, 48) and this investigation, show that they are an important research tool and might help to determine which NPIs are most appropriate for mitigation of viral transmission in different age groups. From the present results, children might be less likely to spread SARS-CoV-2 *via* aerosol transmission and, in addition, seem to be less symptomatic. However, this has to be confirmed in further studies.

This study has several limitations to be considered. Although the PCR testing and exhaled particle count measurements were conducted as close to each other, as possible, they could not be performed simultaneously. Considering the changes in viral load throughout the course of the infection (50), it is possible that the results were affected by this time window. Especially, because only a one-time measurement in each participant was conducted, the dynamics of aerosol emission during an infection is unclear. Edwards et al. (34) found an exponential rise of particle counts in primates up to day 7 of infection, with a steep decline thereafter. Longitudinal measurements are necessary to assess, if this is also true for humans. Furthermore, only asymptomatic and mildly to moderately symptomatic children and adolescents were included in our study. Further studies should assess whether similar quantities of aerosol particles are produced by children and adolescents with more severe infection.

In conclusion, significantly higher counts of exhaled particles could be found in SARS-CoV-2 PCR-positive children and adolescents, when compared to -negative controls. In addition, the median exhaled particle count of all children and adolescents was lower than previous measurements in adults. An improved understanding of exhaled particle counts in various age groups might help to determine feasible and reasonable mitigation strategies for viral transmission.

## Data Availability Statement

The raw data supporting the conclusions of this article will be made available by the authors, without undue reservation.

## Ethics Statement

The studies involving human participants were reviewed and approved by the Ethics Committee of the Goethe University Frankfurt. Written informed consent to participate in this study was provided by the participants’ legal guardian/next of kin.

## Author Contributions

DG, HD, MH, MW, and SZ conceived and designed the trial and SZ was the principal investigator. DG, HD, LH, TL, AL, and EU collected trial data and involved with data curation. DG and MH, were responsible for the formal data analysis. DG, MH, TL, LH, AL, EU, HR, and SZ verified the underlying data supporting the findings of this manuscript. All authors had full access to the full data set in this study and critically reviewed and approved the final version.

## Conflict of Interest

SZ declares receipt of grants or contracts from Böhringer Ingelheim (Germany). EU: Horizon 2020 Erydel in Ataxia, and DLR Projektträger 01KG2030 (Tipp Study). He has received honoraria for presentations from Novartis GmbH, GSK, Vifor Pharma, Böhringer Ingelheim, Lofarma GmbH, Allergopharma GmbH, Allergy Therapeutics, and Sanofi Genzyme. SZ reports personal fees from Aimmune, Novartis GmbH, Böhringer Ingelheim, and IMS HEALTH GmbH & Co. OHG. FW, A-KG, and MW were employed by Palas GmbH. The remaining authors declare that the research was conducted in the absence of any commercial or financial relationships that could be construed as a potential conflict of interest.

## Publisher’s Note

All claims expressed in this article are solely those of the authors and do not necessarily represent those of their affiliated organizations, or those of the publisher, the editors and the reviewers. Any product that may be evaluated in this article, or claim that may be made by its manufacturer, is not guaranteed or endorsed by the publisher.

## Supplementary Material

Supplementary Figure 1 Aerosol Resp-Aer-Meter **(A)** structure and **(B)** function.

Supplementary Figure 2 Sample measurement of Resp-Aer-Meter.
