# Supplementary figures and images for: Exhaled Aerosols in SARS-CoV-2 Polymerase Chain Reaction-Positive Children and Age-Matched-Negative Controls
Source: Front Pediatr. 2022 Jul 18;10:941785. doi: 10.3389/fped.2022.941785 (PMC9339682; doi:10.3389/fped.2022.941785)

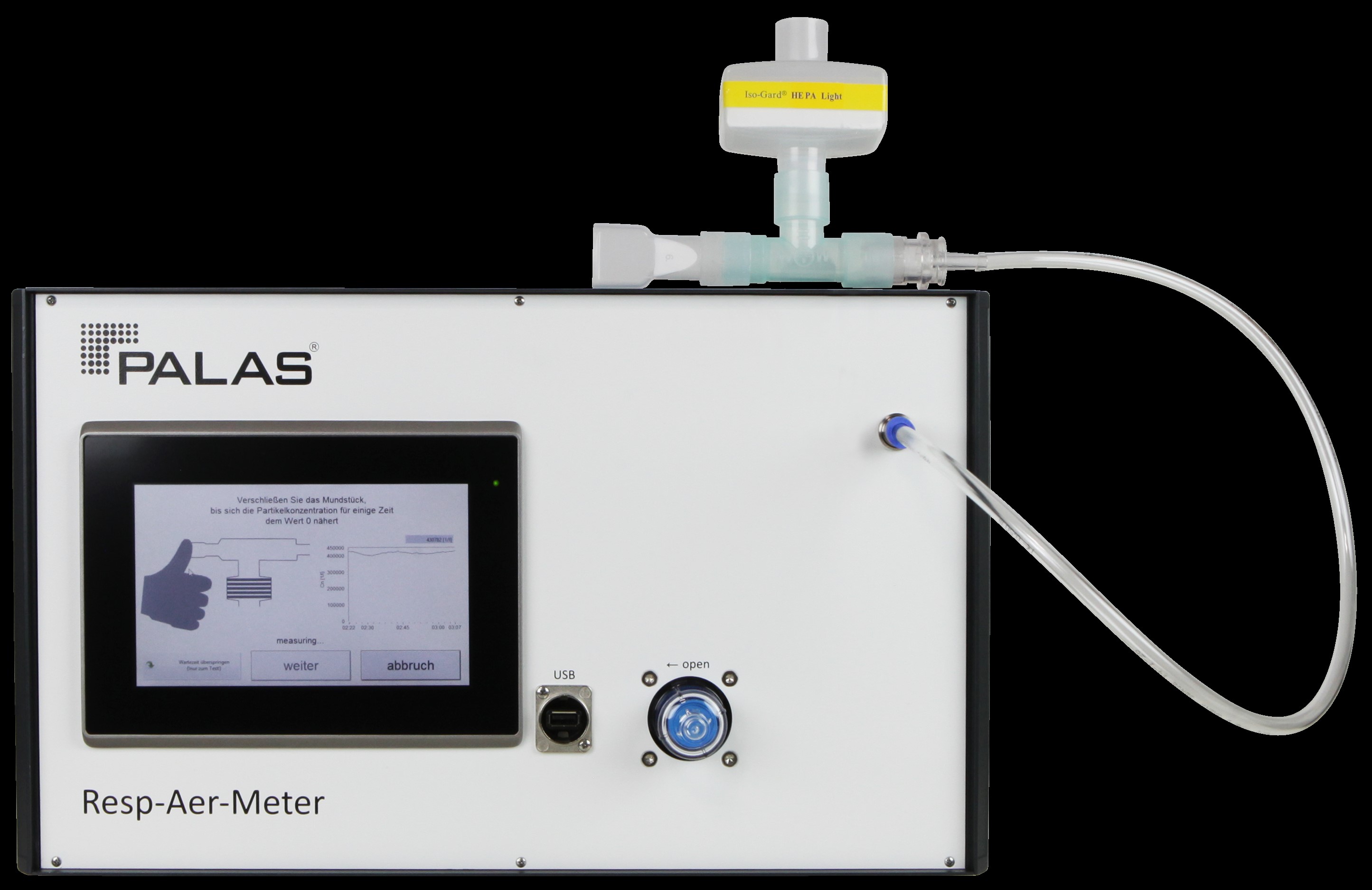

Supplement: Supplementary Figure 1 — Aerosol Resp-Aer-Meter (A) structure and (B) function. [file Image_1.JPEG]

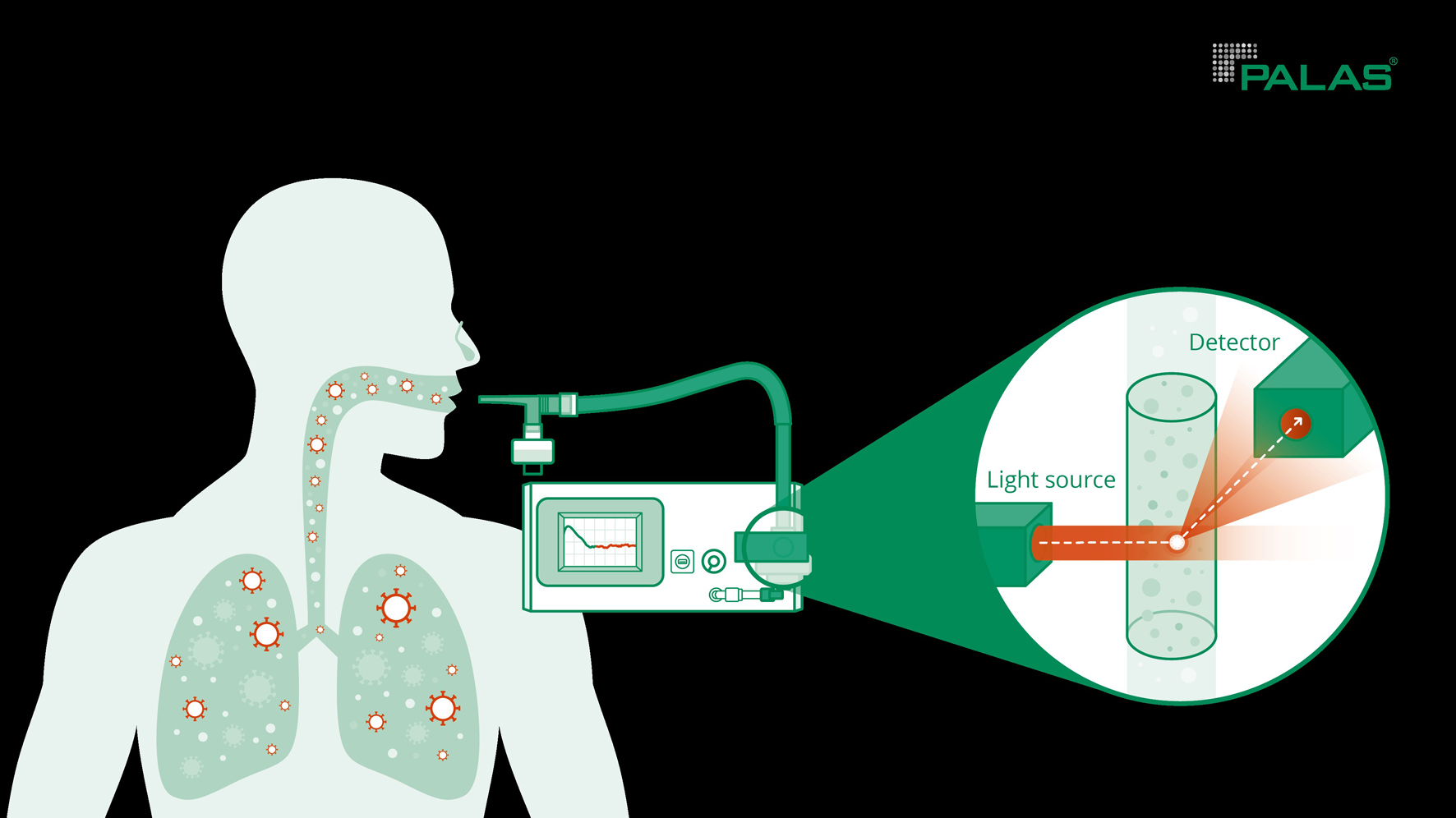

Supplement: Supplementary Figure 2 — Sample measurement of Resp-Aer-Meter. [file Image_2.JPEG]

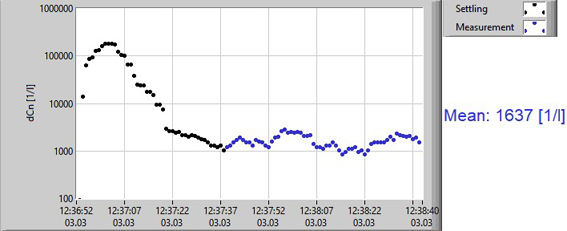

Supplement: Supplementary file 3 [file Image_3.JPEG]
